# Temporal Trends in Perceptions of Anti-tumor Necrosis Factor Risks and Benefits in an Online Community of Patients With Crohn’s Disease

**DOI:** 10.1016/j.gastha.2022.12.007

**Published:** 2022-12-31

**Authors:** Amneet K. Hans, Mark E. Gerich, Blair Fennimore, Frank I. Scott

**Affiliations:** 1 Division of Gastroenterology and Hepatology, Weill Cornell Medicine, New York, New York; 2 Division of Gastroenterology and Hepatology, University of Colorado Anschutz Medical Campus, Aurora, Colorado; 3 Center for Clinical Epidemiology and Biostatistics, Perelman School of Medicine, University of Pennsylvania, Philadelphia, Pennsylvania

**Keywords:** Crohn’s Disease, Biologic Agents, Anti-Tumor Necrosis Factor Agents, Social Media

## Abstract

**Background and Aims:**

Anti-tumor necrosis factor agents (anti-TNFs) have become one of the primary medical therapies for Crohn’s disease (CD). We analyzed perceptions of infliximab and adalimumab in a large online community to better understand the information patients receive.

**Methods:**

Reddit, a vast online community, has several inflammatory bowel disease communities, the largest being /r/CrohnsDisease (rCD), with over 41,000 members. To better understand patient perceptions of biologics, we searched rCD for posts related to “infliximab,” “adalimumab,” and their relevant trade names. The top 20 yearly posts were extracted from 2011 to 2015 and 2011 to 2017, respectively. Manifest coding was performed. Codes were reassessed every 20 posts, resulting in 6 main sentiments. Total codes and per-comment codes were calculated for each sentiment. Percentages for each category were calculated by dividing by the total number of coded sentiments that year. Trends in rates of each sentiment were assessed using Spearman’s correlation coefficients.

**Results:**

4486 comments were analyzed, and 4684 sentiments met our criteria. Negative sentiments decreased for both anti-TNFs over time (infliximab: rho = −0.90, *P* = .04, adalimumab: rho = −0.79, *P* = .04). In our primary analysis, adalimumab injection-related posts increased from 2012 to 2017 (rho = 0.83, *P* = .04). Positive sentiments and sentiments regarding drug costs, loss of efficacy, and diet remained stable. For infliximab and adalimumab, comment volume increased significantly over time (rho 0.90; *P* = .04, rho 0.89, *P* = .01).

**Conclusion:**

Our analysis of a large online community suggests a growing acceptance of biologic therapies among patients with CD over time. These data provide additional insight into the multifaceted framework shaping patients’ perceptions of anti-TNFs.

## Introduction

Inflammatory bowel disease (IBD), which includes Crohn’s disease (CD) and ulcerative colitis, is a chronic, relapsing inflammatory disorder of the gastrointestinal tract. Approximately 1.6 million Americans currently have IBD, with as many as 70,000 new cases diagnosed in the United States annually.^1^ Anti-tumor necrosis factor agents (anti-TNFs) are a major component of medical therapy for CD and include infliximab, adalimumab, and certolizumab. Along with their well-documented clinical efficacy in moderate to severe CD, anti-TNFs also have well described but rare serious risks including lymphoma, nonmelanoma skin cancer, injection or infusion reactions, and severe infections. Patients often weigh these risks in conjunction with the potential benefits of these therapies before making their decision to initiate anti-TNF therapy.^2^

Many individuals with chronic diseases such as IBD engage in online communities to learn more about their disease, explore treatment options, and find support and a sense of community.^3^ In the United States, 77 percent of the population has a social media profile, and this number continues to rise.^4^ A recent survey of patients within the Crohn’s and Colitis Foundation’s Partners cohort found that the majority of patients with IBD spend between thirty minutes and an hour on social media daily, with increased usage associated with active disease.^4^ Among IBD patients, social media offers a venue for patients to share their experiences living with a chronic illness and to give and receive information related to medical therapies such as anti-TNFs.

A 2016 ethnographic study by Frolich and colleagues found that patients with IBD find support through various online communities and use these platforms to redefine what it means to live with their condition.^5^ While the Crohn’s and Colitis Foundation provides a medically affiliated online community forum for patients and their family members to share their experiences and find a support network, other nonmedical social media networking sites such as Facebook, Twitter, and Instagram have become a growing medical resource for patients with IBD as well.^6^ A recent social media survey involving over 1900 patients with IBD revealed that over 51% followed at least 1 and up to 5 IBD-specific social media accounts, and social media use was significantly higher in those with active disease compared to those in remission (odds ratio 0.63; 95% confidence interval, 0.50–0.79).^7^ The majority of patients (56.7%) with IBD spent between 30 minutes to one hour on social media each day.^7^ However, 40% of these patients expressed concern regarding the validity of the content available on these online platforms.

Reddit is one of several popular online forums where individuals can share news and content and comment on other posts.^8^ Launched in 2005, Reddit now contains nearly 1.2 million topic-oriented communities and has an estimated 330 million active users each month.^9^^,^^10^ According to Alexa, an Amazon company that analyzes web traffic, Reddit is now the third-most popular internet destination for users in the United States, surpassing both Facebook and Amazon, and is used by the 18–29 age group the most.^11^^,^^12^ Communities on Reddit are user-created and defined by the topic of discussion. These are called subreddits and are preceded by “/r/”. Reddit has several IBD-related communities, the largest being /r/CrohnsDisease (rCD), which has over 41,000 members.^13^ Unique to Reddit, users in a given subreddit can actively vote on the importance of each post, resulting in a ranking system by the community. This process is defined as “upvoting” or “downvoting,” and provides each post with a specific rank of importance. We analyzed a community that is unmoderated, unlike the Crohn’s and Colitis Foundation, and has a unique user interaction with persistence of users and conversations over time. Unlike other social media platforms, Reddit is not picture-based or transient, allowing us to assess the trends in perceptions of anti-TNFs over time.

One particular area of interest for patients managing IBD relates to the use of biologic therapies, such as anti-TNFs. There are currently limited data on the type of information patients receive from online communities and forums and how patients’ perceptions of anti-TNFs may change over time within these communities. We analyzed rCD, one of the largest online IBD communities, to assess secular trends in perceptions and shared information related to the two primary anti-TNFs used worldwide, infliximab and adalimumab, both of which have been FDA-approved for the treatment of CD since 1998 and 2007, respectively.

## Materials and Methods

**Figure 1:**
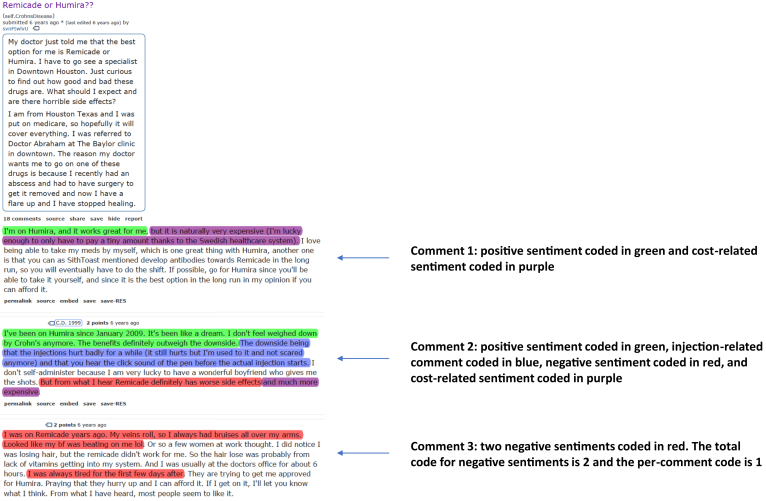
Example of extracted post and relevant coding. Example of the data extracted from rCD. The post subject is followed by 3 comments. Each comment is coded (in different colors) for 6 main sentiments: positive or supportive statements regarding anti-TNFs, negative experiences with anti-TNFs, costs, loss of efficacy, dietary advice, and drug infusion-related/injection-related comments. Total codes for each sentiment and per-comment codes, where each sentiment can be counted for each comment only once, are calculated (see comment 3). anti-TNFs, anti-tumor necrosis factor agents.

To better understand perceptions of biologic therapies, we searched rCD for posts with “infliximab,” “adalimumab,” and their relative trade names in the title. All content on Reddit can be searched anonymously via the use of their publicly available application programming interface. Searches using the Reddit API can also be date-specific and keyword-specific. The 20 top-ranked yearly posts, determined by ranking, were extracted from 2011 to 2015 for infliximab and from 2011 to 2017 for adalimumab. This specific time range was used due to the limited number of biologic-related posts in rCD prior to 2011. For the selected years, infliximab and adalimumab were the most commonly used anti-TNFs which is why the search was performed using the drug names. A more general term such as “biologic” was not utilized as not all biologics are anti-TNF agents. Each of the top 20 yearly posts was evaluated for its individual comments, and each comment was further assessed for 6 main sentiments: positive or supportive statements regarding anti-TNFs, negative experiences with anti-TNFs, costs, loss of efficacy, dietary advice, and drug infusion-related/injection-related comments (Figure 1). Manifest coding of the sentiments was performed. Manifest coding is a type of surface-level analysis that enables researchers to directly identify what the informants say.^14^ Thematic coding, a qualitative method that is useful for reporting themes found within a data set, was developed in an iterative manner: codes were reassessed every 20 posts to identify new emerging themes among the coded sentiments.^15^ The 6 noted sentiments were derived from this process.

Two members of the research team reviewed all posts to ensure coding accuracy (AH and FS). For each year, total codes for each sentiment and per-comment codes, where each sentiment could be counted for each comment only once, were calculated. The same user was able to comment multiple times on a post, and each comment was included in the analysis. Figure 1 provides an example of the data extracted from rCD. In this figure, there is a post, followed by three comments. In comment 1, the positive sentiment, “I’m on Humira, and it works great for me,” is highlighted in green. In comment 3, there are negative sentiments highlighted in red. The total negative sentiment code for this comment is two, and the per-comment negative sentiment code is one. Percentages for each category were calculated by dividing by the total number of coded sentiments that year. Trends in rates of each sentiment were assessed for each year using Spearman’s correlation coefficients. For adalimumab, we performed a sensitivity analysis excluding 2011 given the limited number of comments that year (Table 1).

**Table 2:** Total Comments, Sentiments, and Rates of Categories From 2011 to 2017 for Adalimumab

Year	Total number of comments	Total number of coded sentiments	Sentiments					
			Positive experience/supportive comment	Negative experience/adverse side-effect	Cost	Loss of efficacy	Diet	Drug administration
2011	103	98	27.2%	32.6%	6.5%	4.3%	2.2%	27.2%
2012	419	639	43.1%	17.7%	10.7%	2.9%	4.9%	18.8%
2013	359	482	31.3%	24.2%	4.1%	4.3%	4.1%	26.6%
2014	390	376	25.1%	17.3%	11.5%	0.7%	3.4%	36.3%
2015	458	487	43.4%	19.6%	2.5%	3.4%	2.9%	27.0%
2016	461	422	37.3%	9.9%	16.4%	0.8%	3.8%	28.2%
2017	530	492	33.7%	12.0%	12.2%	2.2%	1.7%	38.0%
Spearman’s rho, *P*-value 2011-2017	rho = 0.89, *P* = .01	rho = 0.29, *P* = .53	rho = 0.29, *P* = .53	rho = −0.79, *P* = .04	rho = 0.50, *P* = .25	rho = −0.58, *P* = .18	rho = −0.36, *P* = .43	rho = 0.64, *P* = .12
Spearman’s rho, *P*-value 2012-2017	rho = 0.83, *P* = .04	rho = −0.14, *P* = .79	rho = 0.03, *P* = .96	rho = −0.65, *P* = .16	rho = 0.54, *P* = .27	rho = −0.37, *P* = .47	rho = −0.83, *P* = .04	rho = 0.83, *P* = .04

## Results

**Figure 2:**
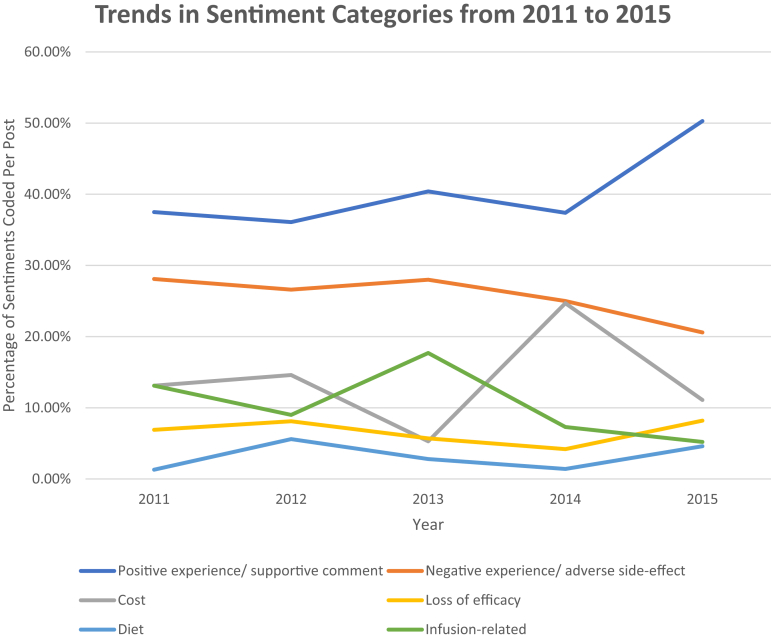
Secular trends in sentiment categories for infliximab from 2011 to 2015.

A total of 4486 comments were analyzed from 2011 to 2017, and 4684 sentiments met our criteria. Regarding infliximab, 1766 comments were analyzed from 2011 to 2015, with 1688 total sentiments meeting the criteria for one of the 6 main sentiments. Among the top 20 posts assessed each year, total comment volume increased significantly from 2011 to 2015 (rho 0.90, *P* = .04), likely due to trends in the numbers of rCD subscribers (Table 2). In our per-comment analysis, sentiments regarding drug costs, infusions, loss of response, and diet were stable over time (Table 2, Figure 2). Positive sentiments slightly increased from 2011 to 2015 time but this was not statistically significant (rho = 0.50, *P* = .39). The percentage of coded negative sentiments regarding biologics decreased significantly over the evaluated period, however (rho = −0.90, *P* = .04).

**Table 1:** Total Comments, Sentiments, and Rates of Categories From 2011 to 2015 for Infliximab

Year	Total number of comments	Total number of coded sentiments	Sentiments					
			Positive experience/supportive comment	Negative experience/adverse side-effect	Cost	Loss of efficacy	Diet	Infusion-related
2011	156	160	37.5%	28.1%	13.1%	6.9%	1.3%	13.1%
2012	380	357	36.1%	26.6%	14.6%	8.1%	5.6%	9.0%
2013	296	282	40.4%	28.0%	5.3%	5.7%	2.8%	17.7%
2014	457	356	37.4%	25.0%	24.7%	4.2%	1.4%	7.3%
2015	477	306	50.3%	20.6%	11.1%	8.2%	4.6%	5.2%
Spearman’s rho, *P*-value	rho 0.90, *P* = .04	rho 0.30, *P* = .62	rho = 0.50, *P* = .39	rho = -0.90, *P* = .04	rho = −0.10, *P* = .87	rho = −0.10, *P* = .87	rho = −0.30, *P* = .87	rho = −0.70, *P* = .19

**Figure 3:**
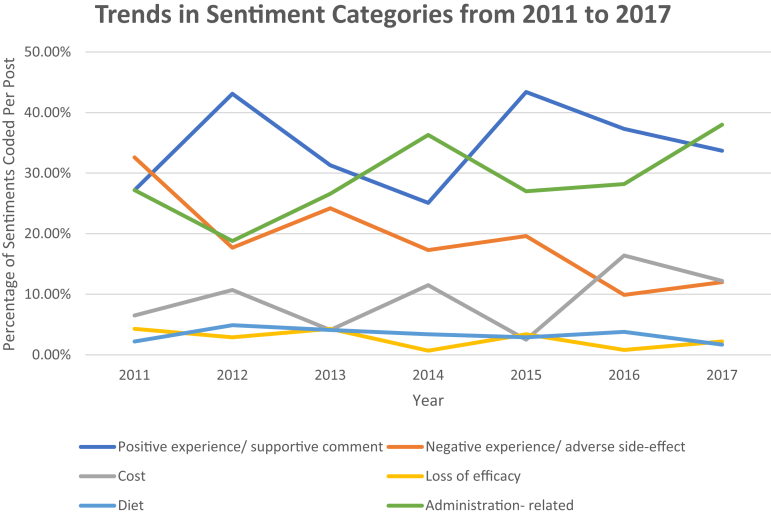
Secular trends in sentiment categories for adalimumab from 2011 to 2017.

Regarding adalimumab, 2720 comments were analyzed from 2011 to 2017, with 2996 coded sentiments (Table 1). From 2011 to 2017, the total comment volume increased (rho 0.89, *P* = .01; Table 1). Negative sentiments decreased over time (rho −0.79, *P* = .04). A substantial number of adalimumab sentiments (28.5%) were related to injection methods. In our primary analysis, injection-related posts increased over time, and this was significant when excluding 2011 (given the low comment volume that year). Positive sentiments and sentiments regarding drug costs, loss of efficacy, and diet remained stable over time (Table 1, Figure 3).

## Discussion

Patients with chronic illnesses such as IBD utilize social networking platforms such as Reddit, Twitter, and Facebook to discuss disease management, explore diagnostic and treatment options, and gather knowledge and support from others with similar experiences. In the United States, 77 percent of the population has a social media profile, and this number continues to rise.^4^ Among IBD patients, social media offers a venue for patients to share their experiences living with a chronic illness and to give and receive information related to medical therapies such as anti-TNFs.

We analyzed the large online community Reddit, and our analysis suggests a growing acceptance of anti-TNF therapies among patients with CD over time. Our manual review of over 4600 sentiments from 2011 to 2017 revealed that comment volume increased during this time, possibly related to increased Reddit subscribers. Negative sentiments decreased for both infliximab and adalimumab, and positive sentiments remained stable. Sentiments regarding cost, loss of efficacy, and diet remained stable.

There are limited data regarding patients’ online perceptions of the risks and benefits of biologic agents. In a smaller study, Martinez and colleagues identified IBD-related posts from Twitter and e-forum discussions from over 3000 social media posts that mentioned biologics and then manually sampled almost 1600 posts that met their criteria.^16^ From their analyses of 452 posts discussing the risks and benefits of biologic agents, they discovered five main themes: negative experiences and concerns, decision-making surrounding their use, positive experiences, information- and support-seeking from the online community, and cost-related discussion. This was one of the first studies to provide insight into patients’ understanding of the risk-benefit profiles of biologic agents and the influence of online communities in shaping patient perceptions outside of direct physician interaction.

We believe our study has several important strengths. To our knowledge, this is the first study to utilize Reddit’s /r/CrohnsDisease, with over 41,000 members, to study patient perceptions related to anti-TNF agents. Furthermore, using these longitudinal data, our study is one of the first to demonstrate how patients’ perceptions of anti-TNFs have changed over time. By utilizing sentiments related to various aspects of anti-TNF therapies, we demonstrated the power of social media analysis to identify additional counseling opportunities for patients within the clinical setting. Although infliximab infusion-related sentiments remained stable, adalimumab injection-related sentiments increased over time suggesting an opportunity for additional counseling for patients regarding the injection itself. In fact, there is now a citrate-free version of adalimumab (approved in 2018) that is associated with less pain following injection given its thinner needle size, smaller volume to administer, and removal of citrate buffers that previously caused pain with injection.^17^ Future analyses of social media posts regarding products may serve manufacturers and allow them to address patient concerns in a more expedited manner.

There are several limitations with regard to this research. Firstly, it is well known that social media attracts attention from the pharmaceutical industry.^18^ Many sponsors within the industry utilize social media platforms such as Facebook, Twitter, and YouTube to commercialize or market their products, share information regarding various diseases, or recruit patients for clinical trials.^18^ Social media has presented itself as yet another opportunity for direct-to-consumer advertising (DTCA). While these practices provide an easy way to deliver product information, DTCA is often unregulated and tends to highlight pharmaceutical drug benefits over risks.^19^ Drug efficacy claims and personal testimonials can be very influential in social media users’ treatment decisions.^19^ Although the direct influence of pharmaceutical companies and their marketing strategies on Reddit users is unclear, it is possible that DTCA has influenced our findings.

There is also the potential for selection bias, both among disease subtype and age group. We analyzed a subreddit focused on CD given its relative size; our findings may not be generalizable to other social media sites, nor to patients with ulcerative colitis. Despite increasing social media use in the United States, there may be patients who do not post on certain social media platforms or patients who do not use social media or have internet access. Social media use may also differ by age; a recent Pew Research Center survey revealed that 88% of 18- to 29-year-olds used any form of social media but this percentage decreased as the patient population got older, with 37% of patients aged 65 and older reporting any social media use.^20^ Although Facebook continues to be the most popular social media platform, with 68% of US adults who are Facebook users, those aged 18–24 were found to be more likely to use other platforms including Snapchat, Instagram, and Twitter.^20^ Furthermore, it is difficult to determine whether some users posted more than others, which may increase selection bias as well.

There is also the potential for misclassification bias. We analyzed the CD forum on Reddit, but there was no way to confirm this diagnosis, so it is possible there may have been disease misclassification. As accounts on Reddit are anonymously created, it is also possible that users could have multiple accounts under different usernames; assessing for confounding due to this is not possible, though similar effects are possible with most other online platforms. Similarly, information bias may have been present due to posts from users misrepresenting who they are. However, this is likely occurring on other online platforms as well. Another limitation to the analysis was addressing a combination of sentiments in a single comment. For example, there could be a negative comment about the cost of medication and it was classified as a cost-related sentiment. Further classification of positive and negative sentiments may have provided a more accurate analysis.

## Conclusion

With the increasing number of biologic agents being used in the management of IBD, patients are increasingly utilizing social media platforms to help inform their perceptions of risk and benefit. Our analysis of the large online community Reddit reveals there may be a growing acceptance of anti-TNF therapies among patients with CD over time. Our study is one of the first to explore how perceptions of anti-TNFs have evolved over time. Overall, there was an increase in comment volume, a decrease in negative sentiments, positive sentiments remained stable, and injection-related sentiments increased for adalimumab. These data provide a framework for better understanding patients’ perceptions of these medications. Further research is required to assess the generalizability of our findings and better understand the information patients are receiving online for CD medications.
